# Visceral Adipose Tissue Inflammatory Factors (TNF-Alpha, SOCS3) in Gestational Diabetes (GDM): Epigenetics as a Clue in GDM Pathophysiology

**DOI:** 10.3390/ijms21020479

**Published:** 2020-01-12

**Authors:** Rebecca C. Rancourt, Raffael Ott, Thomas Ziska, Karen Schellong, Kerstin Melchior, Wolfgang Henrich, Andreas Plagemann

**Affiliations:** 1 Division of ‘Experimental Obstetrics’, Clinic of Obstetrics, Charité—Universitätsmedizin Berlin, Corporate Member of Freie Universität Berlin, Humboldt-Universität zu Berlin, and Berlin Institute of Health, Campus Virchow-Klinikum, 13353 Berlin, Germany; raf.ott@gmail.com (R.O.); thomas.ziska@charite.de (T.Z.); karen.schellong@charite.de (K.S.); kerstin.melchior@charite.de (K.M.); andreas.plagemann@charite.de (A.P.); 2 Clinic of Obstetrics, Charité—Universitätsmedizin Berlin, Corporate Member of Freie Universität Berlin, Humboldt-Universität zu Berlin, and Berlin Institute of Health, Campus Virchow-Klinikum, 13353 Berlin, Germany; wolfgang.henrich@charite.de

**Keywords:** gestational diabetes mellitus, epigenetics, DNA methylation, TNF-α, SOCS3, adipose tissue, mRNA expression

## Abstract

Gestational diabetes (GDM) is among the most challenging diseases in westernized countries, affecting mother and child, immediately and in later life. Obesity is a major risk factor for GDM. However, the impact visceral obesity and related epigenetics play for GDM etiopathogenesis have hardly been considered so far. Our recent findings within the prospective ‘EaCH’ cohort study of women with GDM or normal glucose tolerance (NGT), showed the role, critical factors of insulin resistance (i.e., adiponectin, insulin receptor) may have for GDM pathophysiology with epigenetically modified expression in subcutaneous (SAT) and visceral (VAT) adipose tissues. Here we investigated the expression and promoter methylation of key inflammatory candidates, tumor necrosis factor-alpha (TNF-α) and suppressor of cytokine signaling 3 (SOCS3) in maternal adipose tissues collected during caesarian section (GDM, *n* = 19; NGT, *n* = 22). The mRNA expression of TNF-α and SOCS3 was significantly increased in VAT, but not in SAT, of GDM patients vs. NGT, accompanied by specific alterations of respective promoter methylation patterns. In conclusion, we propose a critical role of VAT and visceral obesity for the pathogenesis of GDM, with epigenetic alterations of the expression of inflammatory factors as a potential factor.

## 1. Introduction

The growing public health problem stemming from the numerous downstream harmful health conditions link to and brought about by overweight and obesity is an important issue in maternal health. The rising prevalence of gestational diabetes mellitus (GDM) is high (>10%) as is overweight and obesity in pregnancy both of which ultimately lead to increased risk for future health complications for mother and child [1,2,3,4,5,6,7,8,9]. The culmination of affluent eating patterns, increased body-mass-index (BMI), and weight gain are key risk factors of developing GDM, which is critically characterized by insulin resistance. 

Visceral obesity, on the other hand, is a key risk factor for overall insulin resistance, e.g., regarding the metabolic syndrome [10,11,12]. Various studies have described a direct relationship between visceral adipose tissue (VAT) and the development of the pre-diabetic condition and diabetes. Mechanistically, this has been critically related to the inflammatory processes within adipose tissue, especially in visceral [13,14]. The particular pathophysiological role and precise mechanisms/factors within VAT for GDM are increasingly of interest.

As obesity is associated with inflammatory changes [15], we wanted to build on our previous investigations/findings on the ‘EaCH’ cohort [16,17,18] and now focused on the inflammatory markers, tumor necrosis factor-alpha (TNF-α) and suppressor of cytokine signaling 3 (SOCS3) which have been identified as key candidates of obesity-related insulin resistance [19,20,21]. Through intensified inflammation comes the promotion of cell death/apoptosis critical in restoring tissue homeostasis. Both TNF-α and SOCS3 are important components in the inflammation and apoptosis processes in the cellular composition of adipose tissue (e.g., adipocytes) [20,21,22,23] and their regulatory mechanism have been previously connected showing TNF-α regulating SOCS3 expression [24]. All of which made investigating these two targets/regulators jointly in maternal adipose tissue of high interest more specifically in GDM, as TNF-α has even been suggested as a potential early risk marker and screening parameter [15,25,26,27].

Epigenetic alterations, especially of promoter methylation patterns, have been proposed as potential causes of insulin resistance, GDM, and obesity in a variety of aspects [2,13,16,17]. Therefore, in order to contribute to a better understanding of GDM pathophysiology, we sought to investigate the potential alterations of both TNF-α and/or SOCS3 mRNA expression and methylation in adipose tissues of women with GDM compared to pregnancies with normal glucose tolerance (NGT). These findings and potential relations were further interrogated with analysis of promoter DNA methylation of both candidates to determine whether this epigenetic modifier influenced/regulated the altered gene expression. 

## 2. Results

### 2.1. Study Cohort

For both study groups (NGT: *n* = 22 and GDM: *n* = 19), maternal and birth characteristics are summarized in Table 1. BMI at both prepregnancy and at the time of delivery as well as maternal age were similar between groups. Both groups were categorized as overweight according to the mean prepregnancy BMI (NGT: 26.8 ± 7.9 kg/m^2^ and GDM: 28.2 ± 6.7 kg/m^2^). For the GDM group, maternal metabolic and hormonal state remained altered at the end of pregnancy as compared to controls (e.g., hyperinsulinemia, hyperglycemia). Women with GDM exhibited higher fasting maternal blood C-peptide, insulin, glucose plasma levels and homeostatic model assessment of insulin resistance (HOMA-IR) compared to the NGT group (Table 1). Maternal plasma TNF-α levels were significantly higher according to GDM (GDM: 0.86 ± 0.35 pg/mL vs. NGT: 0.32 ± 0.35 pg/mL, P < 0.0001), this significance continued after adjusting for BMI (Prepregnancy BMI: P = 0.0001, BMI at delivery: P = 0.0001). Direct relationships were observed between maternal blood TNF-α vs. glucose and C-peptide (Table 1). 

### 2.2. Adipose Tissue Gene Expression Analyses of TNF-α

**Figure 1:**
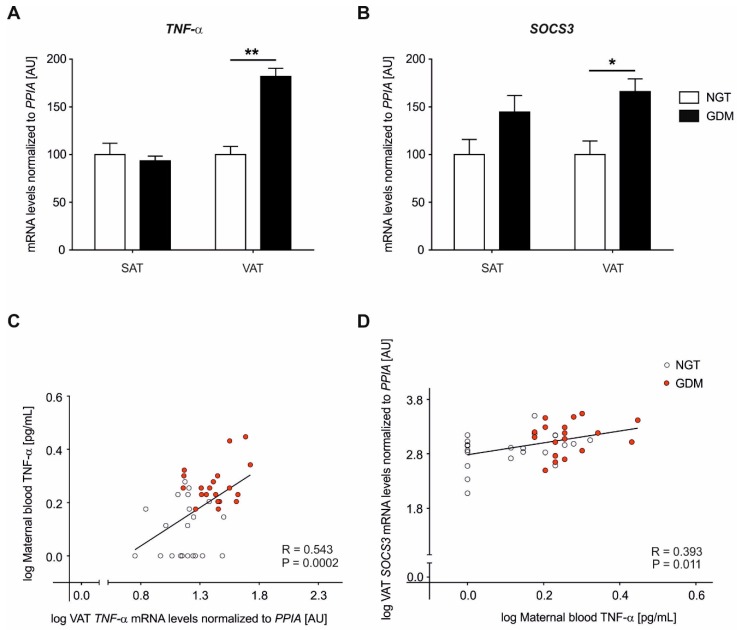
Relative mRNA levels of *TNF*-α and suppressor of cytokine signaling 3 (*SOCS3*) in subcutaneous adipose tissue (SAT) and visceral adipose tissue (VAT) of women with GDM vs. NGT. Relative gene expression of *TNF*-α (**A**) and *SOCS3* (**B**) was normalized to peptidylprolyl isomerase A (*PPIA*) in abdominal SAT and omental VAT, of women with GDM (n = 19, black) vs. NGT women (n = 22, white). Data are means ± SEM, shown as percentage to NGT levels. A.U., arbitrary units. *TNF*-α VAT ** P < 0.0001, *SOCS3*-VAT * P = 0.01. (**C**–**D**) Pearson’s correlation coefficients (R) calculating the relationship between maternal blood TNF-α levels and VAT gene expression data. NGT: open circles, GDM: red circles. Statistical significance between groups (**A**,**B**) and for correlations (**C**,**D**) remained after adjustment for prepregnancy body-mass-index (BMI) and BMI at delivery.

Relative mRNA expression analyses were performed in both subcutaneous adipose tissue (SAT) and VAT for *TNF*-α (Figure 1A). In VAT, mRNA gene expression of *TNF*-α was significantly increased in women with GDM (GDM: 29.1 ± 2.4 vs. NGT: 15.9 ± 1.3, P < 0.0001) while no differences were observed in SAT (Figure 1A). VAT *TNF*-α significance according to GDM status remained after adjustment for BMIs and maternal age (age and prepregnancy BMI P = 0.002, B = 0.225, S.E. = 0.073; age and BMI at delivery P = 0.002, B = 0.212, S.E. = 0.068). This resulted in an overexpression of *TNF*-α mRNA of +82% in VAT of diabetic subjects as compared to controls.

### 2.3. Adipose Tissue Gene Expression Analyses of SOCS3 

*SOCS3* mRNA expression analyses were performed in both adipose tissues, SAT group comparisons showed higher levels in GDM although this was not statistically significant (GDM: 394.6 ± 68.6 vs. NGT: 273.0 ± 43.2, P = 0.091) however, a significant increase in the VAT of women with GDM (GDM: 445.1 ± 61.6 vs. NGT: 267.6 ± 38.2, P = 0.016) was detected (Figure 1B). VAT *SOCS3* significance according to GDM status remained after adjustment for BMIs and maternal age (age and prepregnancy BMI P = 0.037, B = 0.004, S.E. = 0.002; age and BMI at delivery P = 0.034, B = 0.004, S.E. = 0.002). This overexpression of *SOCS3* mRNA in VAT was at +66% for GDM subjects compared to controls. 

### 2.4. Maternal Circulating Plasma TNF-α Levels 

Across the whole cohort (n = 41), maternal circulating TNF-α levels in the blood showed strong correlations with VAT *TNF*-α mRNA expression (Pearson R = 0.543, P = 0.0002, Figure 1C). Additionally, positive associations were observed between maternal blood TNF-α levels and *SOCS3* mRNA expression in VAT (Pearson R = 0.393, P = 0.011, Figure 1D)). The significance of correlations above continued after adjustment for prepregnancy BMI and BMI at delivery. For both genes, no significant correlations were found with maternal blood circulating TNF-α levels and the mRNA gene expression in SAT (*TNF-α*: Pearson R = 0.169, P = 0.300 and *SOCS3:* R = 0.299, P = 0.060). 

### 2.5. DNA Methylation at the TNF-α Promoter in Visceral Adipose Tissue 

**Figure 2:**
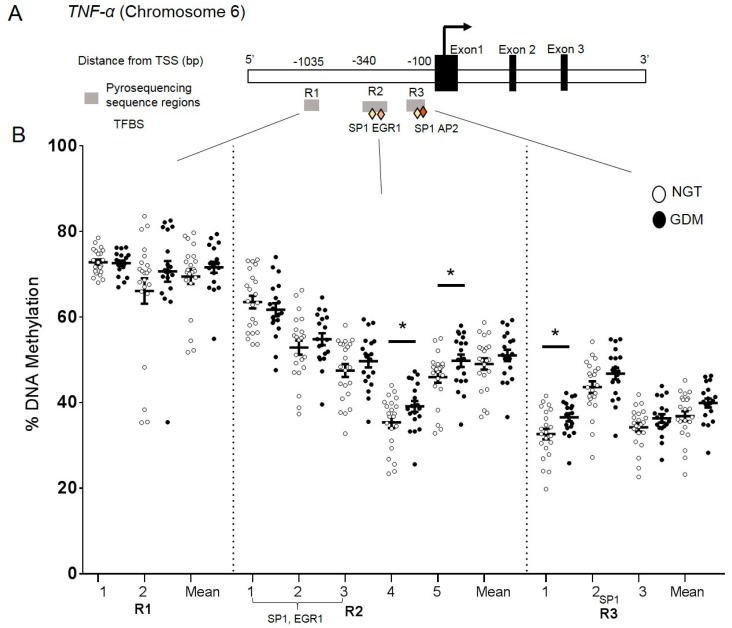
DNA methylation analysis in the *TNF*-α promoter region. CpG site-specific DNA methylation analyses at the *TNF*-α promoter region in visceral adipose tissue from mothers with NGT vs. GDM. (**A**) Schematic illustration of the DNA methylation assays (R1, R2, and R3) for the *TNF*-α promoter region, including transcription factor binding sites (TFBS) Sp1, EGR1, AP2 (diamonds). (**B**) Percent DNA methylation at each individual CpG site investigated (10 CpG sites) in VAT of normal glucose tolerant (NGT; white; n = 22) vs. GDM group (GDM; black; n = 19). Overall mean across CpG sites is also included. Data are means ± S.E.M. * P < 0.05. TSS: Transcriptional start site, bp: basepairs.

DNA methylation analysis was performed in VAT to investigate a possible epigenetic-mechanistic change (according to groups) in the promoter methylation, which could regulate the alterations in gene expression. Three regions (R1, R2, and R3) encompassing 10 CpG sites were investigated (Figure 2A) and the overall DNA methylation levels across the 10 CpG sites ranged from 30–75% at the *TNF*-α promoter (Figure 2B). Small yet significant differences were found between groups at two individual CpG sites in R1 sequence (CpG4; GDM: 39% vs. NGT: 35% P = 0.048 and CpG5; GDM: 49% vs. NGT: 47% P = 0.050) (Figure 2B). CpG1 in R2 region also showed small yet statistically significant differences (CpG1; GDM: 36% vs. NGT: 32% P = 0.018) (Figure 2B). *TNF*-α VAT mRNA expression levels did not significantly correlate with corresponding methylation levels at the individual CpG sites or the overall mean (CpG overall mean: Spearman r = 0.08, P = 0.60). These findings at R1-CpG4, R1-CpG5, and R2-CpG1 were not shown as false positives after correction for false discovery rates (FDR). 

### 2.6. DNA Methylation at the SOCS3 Promoter in Visceral Adipose Tissue

**Figure 3:**
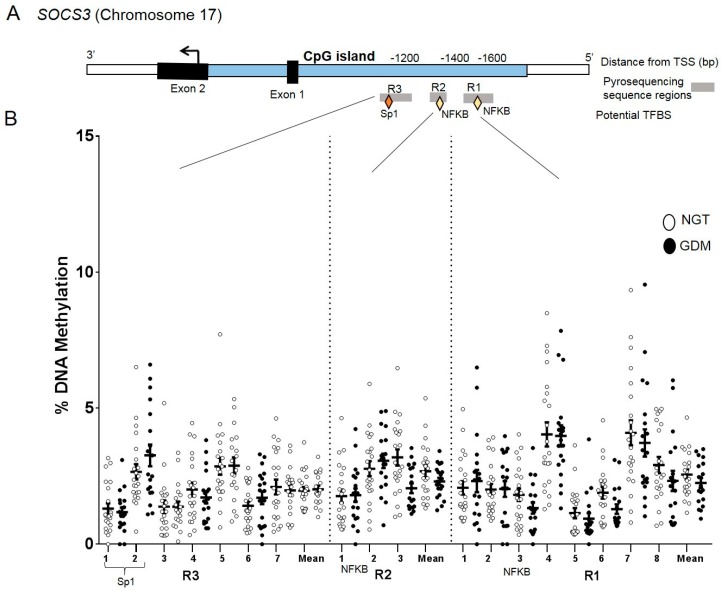
DNA methylation analysis in the *SOCS3* promoter region. CpG site-specific DNA methylation analyses at *SOCS3* within the promoter region in visceral adipose tissue from mothers with NGT vs. GDM. (**A**) Schematic illustration of the DNA methylation assays (R1, R2, and R3) for the *SOCS3* promoter region, including potential transcription factor binding sites (TFBS) Sp1, NFKB (diamonds) and within the CpG island (blue). (**B**) Percent DNA methylation at each individual CpG site investigated (18 CpG sites) in VAT of NGT (white; n = 22) vs. GDM group (GDM; black; n = 19). The overall mean across CpG sites is also included. Data are means ± S.E.M. TSS: Transcriptional start site, bp: base pairs.

In the promoter assays for *SOCS3*, three regions (R1, R2, and R3) covering 18 CpG sites were profiled (Figure 3A). The overall DNA methylation pattern at the *SOCS3* promoter was similar across VAT samples with all 18 CpG sites investigated showing low methylation levels (<5%) (Figure 3B). No significant differences were found between groups at individual CpG sites or with the overall mean (GDM: 2.1% vs. NGT: 2.3%). (Figure 3B). mRNA VAT expression levels did not significantly correlate with corresponding methylation levels (CpG overall mean Spearman r = 0.07, P = 0.65).

## 3. Discussion

We presented the first human study describing both *TNF*-α and *SOCS3* expression in both subcutaneous and visceral adipose tissue in pregnant women affected by overweight and/or GDM, accompanied by promoter methylation analyses of both candidate genes. Both inflammatory factors/markers were found to have increased mRNA expression specifically in VAT, in relation to the GDM phenotype, and independently of the BMI, potentially indicating a specific pathogenic role of VAT-*TNF*-α and VAT-*SOCS3*, respectively for GDM pathophysiology.

*TNF*-α expression is known to be increased in adipose tissue [28,29], especially in VAT. As in previous studies [26,30], we observed elevated circulating maternal (plasma) TNF-α levels in GDM women compared to NGT. Local and/or circulating *TNF*-α stimulates *SOCS3* in adipocytes [21]. Both *TNF*-α and *SOCS3* may increase insulin resistance via differing possible scenarios. One scenario could be a direct interaction of *TNF*-α affecting the insulin receptor [28,31,32,33]. Another is a more indirect scenario by way of *TNF*-α via stimulating *SOCS3* [19,21,24,34] and in turn *SOCS3* through affecting the insulin receptor substrate complex [23,35] leading to the inhibition of the insulin/insulin receptor-mediated pathway, finally resulting in increased adipocyte insulin resistance.

It is worth noting, that both *TNF*-α, as well as *SOCS3*, were found to be significantly increased in VAT of the GDM patients, accompanied by distinct methylation alterations of the *TNF*-α promoter in VAT. However, no significant methylation alterations were observed regarding *SOCS3* nor directly at key transcription factor binding sites (e.g., SP1, NFKB) within the target regions analyzed in both genes. Accordingly, increased *TNF*-α (circulating and/or locally expressed) may regularly activate the *SOCS3* expression, e.g., through the unaffected NFKB binding site at the *SOCS3* promoter, contributing to an increase of *SOCS3* expression. Significant relations (Figure 1D) seem to support this hypothesis. Ultimately, a respective alteration and activated pathway might contribute to adipose tissue insulin resistance and, in turn, overall insulin resistance in GDM. 

Further research into alternative/potential regulatory elements within the gene body of *TNF*-α and regions within the *TNF*-α domain (e.g., tumor necrosis factor receptor 1) could shed insight into the molecular mechanism at play mediating *TNF*-α inflammatory effects on adipocyte function [28]. While methylation has been the most well-studied modifier, other mechanisms such as chromatin modifications (e.g., histones), enhancer and/or RNA elements (e.g., non-coding and miRNAs) need to be further studied as the gene expression changes in *SOCS3* cannot be directly attributed to methylation at the investigated CpG sites presented here. 

The observed alterations specifically in VAT appear to be particularly interesting, in aiming to better understand GDM pathophysiology. The specific, critical role that visceral adipose tissue alterations have in this pathophysiology, have hardly been addressed or not considered in great depth, thus far. However, recent data support the suggestion of an explicit part of VAT and visceral adiposity in GDM [18,36,37,38,39]. Particularly, the expression of insulin receptor and adiponectin were found to be altered in GDM patients, accompanied by altered promoter methylation [16,17]. Importantly, this has been shown to occur even independent of the BMI, which is so far the only adipogenic indicator/marker clinically considered in GDM screening/treatment but, on the other hand, hardly serves as a marker for visceral type of obesity. Reports have purposed that ultrasound could be a potential way of estimating the VAT and SAT risk factors in pregnancy [40,41,42]. In confirmation of the aforementioned observations, our data may point to a particular role visceral obesity might play in the etiopathogenesis of GDM, through altered/increased expression of inflammatory factors and/or further factors increasing insulin resistance through altered action in VAT. 

Finally, limitations and critical aspects of the interpretation should be discussed one of which is that the analyses were on whole tissue samples instead of isolated adipocytes, as in other comparable studies [13,19,36,43] and due to the initially limited sample material available, protein expression analysis could not be performed here. It has been reported that in non-obese humans, macrophages can represent 10–25% of the immune cellular population in VAT, while in an obese condition this range is increased to 40–50% [26,30]. *TNF*-α especially from macrophages, which are enriched in adipose tissue [22,27], might possibly influence the overall expression and protein data of TNF-α. However, clinically and pathophysiologically speaking, it appears rather secondary whether the increased *TNF*-α results directly from adipocytes itself or considerably from an enrichment of macrophages in (increased) adipose tissue, respectively. Despite this, we remain confident in our findings of the link to GDM considering both study groups are well-matched with BMI and on average are categorized as overweight. Clinically, the question of the cell-specific origin does finally not decisively matter on/affect the critical role of increased *TNF*-α for visceral adipose tissue insulin resistance, the more so when additionally considering the increased circulating TNF-α plasma levels generally resulting from adiposity, which are, as shown here, particularly linked with increased VAT expression, specifically. However, to even more concretely understand the pathophysiology behind, future studies should aim to better differentiate the cells of origin of increase *TNF*-α, i.e., by using microdissection of adipose tissues for cell type-specific analyses.

In summary and conclusion, both the increased VAT expression and circulating TNF-α protein, as well as the accompanying increased *SOCS3* expression in VAT, appear to be related to GDM, even irrespective of the BMI. Altered promoter methylation in VAT might contribute to *TNF-*α increase. These findings speak of a specific role visceral adipose tissue and affecting inflammatory processes might play in the pathogenesis of GDM. This should be addressed in more depth in future studies, to improve understanding of and, in turn, screening and treatment of GDM. Understanding how adipose tissue (especially visceral) acts as a diseased organ specifically in the inflammatory pathways should enable more strategic development for prediction, prevention and possible treatment measures, especially concerning GDM.

## 4. Materials and Methods

### 4.1. Subject Data

The research presented here is part of the prospective observational ‘Early CHARITÉ (EaCH)’ cohort study [16,17,19,44]. Genes involved in the inflammatory process were further investigated on the cases in which the optimal material and factors (such as transcription, metabolic and hormonal) could be measured in a complete set so as to avoid a missing data bias. Nineteen women with GDM and 22 women with NGT were prospectively recruited prior to their scheduled Cesarean section (CS) of singletons at the Clinic of Obstetrics of the Charité – Universitätsmedizin Berlin, Campus Virchow–Klinikum, Germany. Standardized procedures/methods, recruitment, exclusion criteria, analytical approaches etc. are described elsewhere in detail [44]. The study groups were matched for maternal age, socio-economic status (SES), ethnic origin, parity and specifically, prepregnancy BMI. BMIs were calculated with maternal height and weight prior to conception and the last measured weight within one week before delivery. BMIs were categorized according to the WHO criteria (normal weight: 18.5–24.9 kg/m^2^, overweight: 25.0–29.9 kg/m^2^, obese: ≥30.0 kg/m^2^). GDM screening was performed between gestational weeks 24 to 28 according to the national guidelines at the time of recruitment [45,46]. No oral antidiabetic drugs were administered in the GDM group, eight were treated by diet and eleven were treated with diet and additional insulin therapy to achieve glycemic control. Further clinical parameters such as plasma insulin, C-peptide, glucose and HOMA-IR were determined for these cases as described elsewhere [16,17,44,47]. The research design and methodology were conducted in accordance with the Declaration of Helsinki, revised in 2004, and approved by the local Ethics Committee (Ethikausschuss 2 am Campus Virchow–Klinikum, Charité Universitätsklinikum Berlin, EA2/026/04). Informed written consent was obtained by all participants before inclusion in the study. 

### 4.2. Maternal Blood and Adipose Tissue Sampling

Fasting maternal venous blood was collected prior to the planned Cesarean section (CS). Plasma and blood cell fractions were stored at −80 °C for further analysis. Paired maternal biopsies of visceral adipose tissue (VAT) from the greater omentum and subcutaneous adipose tissue (SAT) from the abdominal anterior wall were obtained during planned CS delivery. Samples were immediately snap-frozen in liquid nitrogen and stored at −80 °C. 

### 4.3. Maternal Blood TNF-α Analysis

Total plasma TNF-α was determined using a specific commercially available Ultrasensitive Human-TNF-alpha-ELISA (Cat# KHC3013, Invitrogen Corporation, Carlsbad, CA, USA). Assays were performed using 100 µL of plasma according to the manufacturer’s protocol. The intra- and inter-assay coefficients of variations were: 3.9–5.2% and 5.9–8.5% respectively in a concentration range of 47.0–459.0 pg/mL. 

### 4.4. Gene Expression Analyses

Total RNA was isolated from adipose tissue (100 mg) using the RNeasy Lipid Tissue Mini Kit (Qiagen, Hilden, Germany) according to the manufacturer’s protocol. Assessment of purity and quantity was measured with both a spectrophotometer (NanoDrop 1000, Thermo Scientific, Wilmington, DE, USA) and with a Bioanalyzer 2100 (Agilent Technologies, Santa Clara, CA, USA). A starting sample of 300 ng of RNA was reverse transcribed for each case with the iScript kit (Bio-Rad, Hercules, CA, USA). Using TaqMan technology (Applied Biosystems, Waltham, MA, USA), quantitative real-time PCR was performed in triplicate on a 7500 instrument (Applied Biosystems) with the respective controls and quality checks included. Pre-designed exon-exon spanning TaqMan primer assays from Applied Biosystems were used (ID: *TNF-α*: Hs01113624_g1; *SOCS3*: Hs02330328_s1) and amplified in singleplex with the housekeeping gene peptidylprolyl isomerase A (*PPIA*: Hs99999904_m1) [48]. Gene expression was normalized using the 2−ΔCt method, including correction for amplification efficiency calculated from the standard curves for each primer set [49,50]. As in previous reports [16,17,18], gene expression of PPIA showed no group differences in both VAT and SAT. 

### 4.5. DNA Methylation Analyses

Genomic DNA was extracted from VAT (30 mg starting material in total), using the Genomic DNA-Tissue kit and the Quick-gDNA Blood kit (Zymo Research, Irvine, CA, USA), according to manufacturer’s protocols. Bisulfite conversions were performed using the EZ DNA Methylation Gold kit (Zymo Research). The target promoter regions were selected according to literature and/or in silico analyses of genomic elements such as CpG islands and transcription factor binding sites within the sequences [34,51,52,53]. The investigated chromosomal locations for the TNF-α promoter region was chr6:31,542,413-31,543,347 and for the *SOCS3* promoter region was chr17:76,356,234-76,356,871 according to the UCSC Genome browser on the human February 2009, GRCh37/hg19 assembly. Methylation assays were designed with PyroMark Assay Design Software v. 2.0 (Qiagen). Primer and assay information is available in supplemental Table S1. Pyrosequencing was performed on amplified PCR products using the PyroMark Q24 pyrosequencer (Qiagen). Percent methylation was analyzed across individual measured CpG sites (10 CpG sites, *TNF-α*; 18 CpG sites, *SOCS3*). The reproducibility and specificity of the different assays were tested and validated with duplicate samples, various tissue types, and methylation scales (0–100%). 

### 4.6. Statistical Analyses

Data are presented as means ± SD, means ± SEM or number and percentage. Gene expression data are presented as arbitrary units. Group comparisons were analyzed by unpaired *t*-test or Mann-Whitney-U-test as appropriate. Normal distribution of continuous variables was evaluated by Shapiro-Wilk-tests and skewed data were transformed logarithmically to achieve normal distribution. Spearman’s correlation coefficients (r) were calculated to assess associations between clinical and/or endocrine parameters, gene expression and DNA methylation, respectively. Pearson’s correlations coefficients (R) were used to test the relationship between maternal TNF-α plasma levels and gene expression. Potential confounding effects of maternal BMI (prepregnancy and at delivery) and maternal age were checked using partial Pearson’s correlation. Binary logistic regression analysis was run to evaluate associations between mRNA expression and GDM adjusting for BMIs (prepregnancy and at delivery) and maternal age as covariates. For methylation analyses, CpG sites were additionally run through a two-stage step-up method of Benjamini, Krieger, and Yekutieli to correct for FDR and to ensure that the statistically significant findings were discoveries and not false positives [54]. Statistical analyses were performed with GraphPad Prism 7.00 (GraphPad Software, San Diego, CA, USA) and SPSS 23.0 software (IBM, Munich, Germany). Statistical significance was set at P < 0.05.

## Figures and Tables

**Table 1:** General and specific cohort characteristics according to groups, gestational diabetes mellitus (GDM) and normal glucose tolerance (NGT) [18], and relations with maternal blood tumor necrosis factor alpha (TNF-α) at delivery.

	NGT *n* = 22 [18]	GDM *n* = 19	*p*-Value *	Spearman’s vs. MB TNF-α r (*p*-Value *)
Maternal age (years)	32.0 ± 5.3	32.5 ± 4.2	0.72	0.043 (0.78)
Prepregnancy BMI (kg/m^2^)	26.8 ± 7.9	28.2 ± 6.7	0.32	0.107 (0.50)
BMI at delivery (kg/m^2^)	33.1 ± 9.2	33.2 ± 6.6	0.50	0.099 (0.53)
Blood glucose at oGTT (mg/dL)				
Fasting	79.5 ± 8.1	100 ± 30.9	<0.0004	0.380 (0.01)
1-h	120.9 ± 29.3	213 ± 36.8	<0.0001	0.505 (0.0007)
2-h	90.3 ± 19.9	167 ± 49.5	<0.0001	0.516 (0.0006)
Area under the curve (mg/dL*h)	205.8 ± 38.3	346 ± 71.9	<0.0001	0.516 (0.0006)
Maternal fasting plasma levels at delivery:				
Glucose (mg/dL)	71.1 ± 10.7	82.8 ± 8.4	0.001	0.319 (0.04)
Insulin (µU/mL)	21.5 ± 16.1	40.9 ± 36.8	0.05	0.111 (0.48)
HOMA-IR	3.2 ± 1.3	8.4 ± 7.4	0.006	0.208 (0.19)
C-peptide (ng/mL)	2.0 ± 0.8	5.0 ± 3.1	<0.0001	0.338 (0.03)
TNF-α (pg/mL)	0.32 ± 0.35	0.86 ± 0.35	<0.0001	n.a.
Infant parameters:				
Birth weight (g)	3365 ± 495.9	3585 ± 454.4	0.06	0.064 (0.68)
Relative Birth weight (g/cm)	66.4 ± 6.8	70.16 ± 8.1	0.10	0.029 (0.85)

Data are means ± SD, * Statistically significant (*p*-value < 0.05). Normal glucose tolerance, (NGT); gestational diabetes mellitus, (GDM); maternal blood, (MB); body-mass-index, (BMI); Oral glucose tolerance test, (oGTT); homeostatic model assessment of insulin resistance, (HOMA-IR); tumor necrosis factor alpha, (TNF-α); not applicable, (n.a.). Statistically significant differences between groups and correlations remained after adjustment for prepregnancy BMI and BMI at delivery.

## Supplementary Materials

Supplementary materials can be found at https://www.mdpi.com/1422-0067/21/2/479/s1. Supplementary Table S1: Primer information for DNA methylation assays: *TNF*-α and *SOCS3*.

## Abbreviations

BMI	Body-mass-index
CS	Cesarean section
GDM	Gestational diabetes mellitus
HOMA-IR	Homeostatic model assessment of insulin resistance
NGT	Normal glucose tolerance
oGTT	Oral glucose tolerance test
PPIA	Peptidylprolyl isomerase A
SAT	Subcutaneous adipose tissue
SOCS3	Suppressor of cytokine signaling 3
TNF-α	Tumor necrosis factor alpha
VAT	Visceral adipose tissue
