# Supplementary material for: Visceral Adipose Tissue Inflammatory Factors (TNF-Alpha, SOCS3) in Gestational Diabetes (GDM): Epigenetics as a Clue in GDM Pathophysiology
Source: Int J Mol Sci. 2020 Jan 12;21(2):479. doi: 10.3390/ijms21020479 (PMC7014132; doi:10.3390/ijms21020479)
Supplement: Supplementary file 1 [file ijms-21-00479-s001.pdf]

Supplementary Table S1. Primer information for DNA methylation assays

Methylation assay	Chromosomal location*	Primer information (5′ to 3′)		PCR information	
		Orientation	Bisulfite converted sequence	Amplicon size (bp)	Annealing temp. (°C)
TNF-α R1	chr6:31,542,413-31,542,518	Forward	TGTGATTATAGTAATGGGTAGGAGAATGTT	105	52
		Reverse†	TTCACTCCCTAAAACCCTCTACATAAC		
		Sequencing 1	GAATGTTTAGGGTTATGG		
TNF-α R2	chr6:31,543,125-31,543,346	Forward	AGG GGT ATT TTT GAT GTT TGT GTG	222	52
		Reverse†	CTA CTA ACT AAA TAT ACC AAC AAC TACC		
		Sequencing 1	GAT GTT TGT GTG TTT TTA ATT TTT TAA AT		
TNF-α R3	chr6:31,543,123-31,543,347	Forward	GAGGGGTATTTTTGATGTTTGTGTG	223	52
		Reverse†	CTACTAACTAAATATACCAACAACTACC		
		Sequencing 1	ATGGGTTTTTTTATTAAGGAAGTT		
SOCS3 R1	chr17:76,356,492-76,356,871	Forward	AGGGTTGGTAAAGAATTTGG	379	50
		Reverse†	CCCTCCCTTCTAAAAAAACTAATTT		
		Sequencing 1	TTTAGGTAGGTTTTTTAGAATTGT		

SOCS3 R2	chr17:76,356,492-76,356,871	Forward	AGGGTTGGTAAAGAATTTGG	379	50
		Reverse†	CCCTCCCTTCTAAAAAAACTAATTT		
		Sequencing 1	GTTGGTAAAGAATTTGGTAG		
SOCS3 R3	chr17:76,356,234-76,356,484	Forward	AAGTGTGAATGAGAAGTTG	251	50 ZymoTaq
		Reverse†	ACCTTCTTATAATATTTAATCACTACT		
		Sequencing 1	GTGTGAATGAGAAGTTG		

bp, base pairs. CpG, Cytosine followed by guanine nucleotide. *According to UCSC Genome browser on human Feb. 2009, GRCh37/hg19 assembly. †Biotinylated primer.
